# A Possible Genetic Link between MTHFR Genotype and Smoking Behavior

**DOI:** 10.1371/journal.pone.0053322

**Published:** 2012-12-28

**Authors:** Michael Linnebank, Susanna Moskau, Alexander Semmler, Barbara Hoefgen, Gisela Bopp, Ulf Kallweit, Wolfgang Maier, Christian G. Schütz, Ullrich Wüllner

**Affiliations:** 1 Department of Neurology, University Hospital Bonn, Bonn, Germany; 2 Department of Psychiatry, University Hospital Bonn, Bonn, Germany; 3 Department of Neurology, University Hospital Zurich, Zurich, Switzerland; 4 Institute of Mental Health, University of British Columbia, Vancouver, Canada; Idaho State University, United States of America

## Abstract

**Background:**

Hyperhomocysteinemia is an independent risk factor for stroke and other vascular events. The variant methylenetetrahydrofolate reductase (MTHFR) C677T is associated with elevated homocysteine levels, cardiovascular disease and stroke, which supports a causal relationship between hyperhomocysteinemia and vascular disease. However, MTHFR variants have also been reported to be associated with smoking behavior, which could be an important confounder.

**Methodology/Principal Findings:**

We analyzed the MTHFR variants C677T and A1298C in two independent samples of 525 and 535 individuals, respectively. 21% of the non-smokers, but only 12% of the smokers were homozygous carriers of both MTHFR wildtype alleles, i.e. 677CC and 1298AA (Chi^2^ = 15.8; p<0.001; binary regression). Plasma homocysteine levels were higher in smokers (13.9±4.1 µmol/L) than in non-smokers (12.6±4.0 µmol/L; F = 11.4; p = 0.001; ANOVA). Smoking MTHFR 677TT individuals had the highest plasma homocysteine levels (16.2±5.2 µmol/L), non-smoking 677CC individuals had the lowest (12.2±13.6 µmol/L).

**Conclusions/Significance:**

In our study samples, MTHFR variants and smoking behaviour were associated with homocysteine plasma levels. In addition, the MTHFR variants were associated with smoking behaviour. Such an association may be a relevant confounder between MTHFR variants, homocysteine plasma levels and vascular diseases.

## Introduction

Hyperhomocysteinemia is an independent risk factor for stroke and other vascular events (OMIM 603174). The association of the T-allele of MTHFR C677T (A222V) with hyperhomocysteinemia, cardiovascular disease and stroke suggests a causal relationship between hyperhomocysteinemia and vascular disease, and a meta-analysis stated evidence for causality from Mendelian randomisation, arguing that the observed increase in the risk of stroke among individuals homozygous for the T-allele is close to that predicted from the differences in homocysteine concentration conferred by this variant [1]. However, the editorial comment on that article pointed out, that the groups defined by the MTHFR C677T genotypes “*should not differ systematically in any other way. For example, those with the TT genotype should not be more likely to be smokers … than individuals with the CC genotype*”[1]. Such an association would be a possible important confounder between MTHFR, homocysteine plasma levels, smoking and vascular events like stroke. A few studies investigated such linkage of the MTHFR genotype with smoking behaviour. In 2001, a study including 232 Hispanic pregnant women revealed a significantly higher rate of smoking (0.59) in TT homozygotes compared with heterozygotes (0.21) or CC homozygotes (0.34). Re-calculation of this data reveals a higher rate of the TT genotype in smokers (0.15) than in non-smokers (0.04) [2]. Newer and larger studies did not provide conclusive data whether smoking is associated with the MTHFR C677T variant. For example, Brown et al. (n = 407) observed a TT genotype frequency of 0.16 in smokers and of 0.12 in non-smokers (not significant) [3]. Liu et al. (n = 1074) reported a higher frequency of the TT genotype in smokers (0.17) than in non-smokers (0.11), but this difference was not statistically tested [4], Husemoen et al did not observe an association of the C677T genotype with daily smoking in 2788 Danish men and women aged 30–60 years [5]. In a small population, Imyanitov et al. reported a higher frequency of homozygosity for the wild-type allele of another MTHFR missense variant, i.e. A1298C, in non-smokers versus smokers, but this was not tested for significance. The C677T genotype was without difference [6]. The two mentioned MTHFR variants, C677T and A1298C, are in linkage disequilibrium, i.e. the haplotype with the two mutant variants 677T;1298C is rare [7]. In addition to possible genetic associations, smoking is associated with increased homocysteine plasma levels, and smoking and MTHFR C677T may interact concerning the association with homocysteine plasma levels [3]. In this study, we aimed at analysing the association of MTHFR genotypes with smoking in two independent samples of 525 and 535 individuals.

## Methods

### Objectives

The available data on the association of MTHFR C677T and A1298C with smoking behaviour is inconclusive. We aimed at analysing MTHFR genotypes and smoking behaviour and their possible interaction regarding homocysteine plasma levels in further populations. As the MTHFR enzyme is a homodimer, missense variants on different alleles in the sense of combined heterozygosity may lead to relevant interactions on the protein level, as in a mixed dimer, amino acid exchanges on one of the strains may influence the other [8]. Thus, we decided to analyse MTHFR C677T; A1298C haplotypes and genotypes instead of analysing these two variants separately to test the hypothesis that the MTHFR genotype defined by C677T and A1298C is associated with smoking behaviour.

### Participants

We analyzed two independent samples, 1060 individuals in total (table 1). Sample 1 (n = 525) consisted of serial in- and outpatients of the division of ultrasonography of the Department of Neurology of the University Hospital Bonn in addition to patients' partners recruited within an ongoing study [2]. Mean age was 48.8±16.0 years, 271 individuals were female, 254 male. Smoking status was defined by self-reported current smoking status, categorized as either smokers or non-smokers.

**Table 1:** Demographic data.

	sample 1	sample 2	all
	525	535	1060
smokers n	311	388	699
smokers: ratio female	0.42	0.48	0.45
smokers: mean age	48.4y±15.2	41.6±12.8	47.0±15.0
non-smokers n	214	147	361
non-smokers: ratio female	0.66	0.40	0.55
non-smokers: mean age	49.4±17.2	35.8±11.6	43.9±16.6

Sample 2 was recruited from an ongoing smoking cessation study run by the Department of Psychiatry, University Hospital Bonn: 388 serial current smokers living in the area of Bonn and 147 population controls, i.e. never-smokers. Mean age was 40.0±12.5 years, 245 individuals were female, 290 male. Status “current smoking” was defined by smoking a minimum of 15 cigarettes per day for more than two years. In both samples, only unrelated individuals of Caucasian ancestry were enrolled.

### Description of Procedures or Investigations undertaken

Total homocysteine plasma levels were analyzed in the clinical routine in the University Hospital Bonn for sample 1, but were not available for sample 2. MTHFR C677T and A1298AC were analyzed by polymerase chain reaction amplification with subsequent allele-specific endonuclease restriction and agarose gel electrophoresis as published [3]. For quality control, each 5^th^ sample was analysed twice. We aimed to analyse the association of the variant MTHFR genotypes with smoking behaviour. As strategy for analysis, we made use of the linkage disequilibrium of MTHFR C677T and A1298C: Because of the linkage disequilibrium, only three MTHFR C677T;A1298C haplotypes occur with relevant frequency: 677C;1298A, 677C;1298C and 677T;1298A.

Thus, we grouped the resulting genotypes:

1. wild-type for both alleles (677CC;1298AA),
2. homozygous for one mutant variant (677TT;1298AA or 677CC;1298CC),
3. heterozygous for one or both mutant variants (.677CT;1298AA or 677CT;1298AC or 677CC;1298AC) [3].

### Statistical methods

Genotype distributions were tested by Chi^2^ goodness-of-fit test. Due to differences of age and gender between smokers and non-smokers, logistic regression models adjusted for age and sex with smoking status as dependent variable were used for statistical analyses. For the analyses of homocysteine plasma levels as dependent variable and MTHFR genotypes, smoking status, age and gender as covariables, multivariate linear regression analysis was used. Due to the different recruitment strategies and criteria, samples 1 and 2 were analyzed separately in addition to a pooled analysis. Homocysteine plasma levels were exploratively compared between groups defined by MTHFR variants of smoking behaviour using ANOVA.

### Ethics

This study has been conducted according to the principles expressed in the Declaration of Helsinki. The study was approved by the local ethics committee (Ethikkommission an der Medizinischen Fakultät der Universität Bonn). All patients gave written informed consent.

## Results

Genotyping succeeded for all DNA samples. The genotype distribution did not deviate from the Hardy-Weinberg equation.

The wild-type genotype 677CC; 1298AA was more common in non-smokers than in smokers in sample 1 (Chi^2^ = 7.6; p = 0.022), in sample 2 (Chi^2^ = 6.0; p = 0.051 for trend) and in the pooled sample (Chi^2^ = 15.8; p<0.001; table 2).

**Table 2:** MTHFR variants in smokers and non-smokers.

all individuals (samples 1+2; n = 1060)				
**c.677C>T**	CC	CT	TT	nominal regression
smokers (n = 699)	0.41	0.47	0.12	Chi^2^ = 5.5; p = 0.064
non-smokers (n = 361)	0.49	0.42	0.09	
**c.1298A>C**	AA	AC	CC	
smokers (n = 699)	0.46	0.43	0.11	Chi^2^ = 6.4; p = 0.040
non-smokers (n = 361)	0.53	0.38	0.10	
**c.677C>T;c.1298A>C**	c.677CC;c.1298AA	c.677TT;c.1298AA or c.677CC;c.1298CC	all others	
smokers (n = 699)	0.12	0.22	0.66	Chi^2^ = 15.8; p<0.001
non-smokers (n = 361)	0.21	0.19	0.60	

sample 1; n = 525				
**c.677C>T**	CC	CT	TT	nominal regression
smokers (n = 311)	0.44	0.46	0.09	Chi^2^ = 3.7; p = 0.159
non-smokers (n = 214)	0.50	0.42	0.07	
**c.1298A>C**	AA	AC	CC	
smokers (n = 311)	0.45	0.45	0.10	Chi^2^ = 4.3; p = 0.116
non-smokers (n = 214)	0.53	0.40	0.07	
**c.677C>T;c.1298A>C**	c.677CC;c.1298AA	c.677TT;c.1298CC or c.677CC;c.1298CC	all others	
smokers (n = 311)	0.14	0.19	0.67	Chi^2^ = 7.6; p = 0.022
non-smokers (n = 214)	0.23	0.15	0.62	

sample 2; n = 535				
**c.677C>T**	CC	CT	TT	nominal regression
smokers (n = 388)	0.39	0.47	0.14	Chi^2^ = 4.7; p = 0.095
non-smokers (n = 147)	0.48	0.41	0.12	
**c.1298A>C**	AA	AC	CC	
smokers (n = 388)	0.47	0.41	0.11	Chi^2^ = 2.9; p = 0.234
non-smokers (n = 147)	0.52	0.35	0.13	
**c.677C>T;c.1298A>C**	c.677CC;c.1298AA	c.677TT;c.1298CC or c.677CC;c.1298CC	all others	
smokers (n = 388)	0.10	0.24	0.66	Chi^2^ = 6.0; p = 0.051
non-smokers (n = 147)	0.18	0.24	0.58	

Genotype frequencies concerning the two alleles MTHFR c.6777C>T and MTHFR c.1298A>C as well as the genotype frequencies resulting from the c.677C>T;c.1298A>C haplotypes.

The T-allele of MTHFR 677C>T was associated with higher homocysteine levels showing a gene-dose effect (B = 1.0; p<0.001; Table 3). In addition, homocysteine levels were higher in smokers (13.9±4.1 µmol/L) than in non-smokers (12.6±4.0 µmol/L; F = 11.4; p = 0.001; ANOVA). Stratification by smoking behaviour and MTHFR C677T revealed an interaction between these two factors on homocysteine plasma levels: smokers and homocysteine plasma levels (C677T genotype): 13.4±4.2 µmol/L (CC), 13.9±3.8 µmol/L (CT), and 16.2±5.2 µmol/L (TT), and non-smokers: 12.2±13.6 µmol/L (CC), 12.7±4.0 µmol/L (CT), and 14.4±4.0 µmol/L (TT). Thus, smoking MTHFR 677TT individuals had the highest plasma homocysteine levels, non-smoking 677CC individuals the lowest.

**Table 3:** Homocysteine levels and MTHFR variants.

**c.677C>T**	CC	CT	TT	linear regression
homocysteine	12.9±4.0	13.5±3.9	15.5±5.4	B = 1.0; p<0.001
**c.1298A>C**	AA	AC	CC	linear regression
homocysteine	13.5±4.3	13.2±4.1	13.3±3.7	B = 0.01; p = 0.944
**c.677C>T;c.1298A>C**	MTHFR c.677CC & MTHFR c.1298AA	all others	MTHFR c.677TT or MTHFR c.1298CC	linear regression
homocysteine	12.4±3.7	13.4±4.0	14.4±4.7	B = 0.8; p = 0.008

Fasting total plasma homocysteine levels in µmol/l ± one standard deviation.

## Discussion

The mutant alleles of the variants MTHFR C677T and, with less evidence, A1298C are associated with both elevated homocysteine plasma levels and vascular diseases arguing for hyperhomocysteinemia as a causal risk factor for vascular disease [1]. However, there are inconclusive data that these MTHFR variants may questionably also be associated with smoking behaviour [2], [3], [4], [5], [6]. Such an association would be an important confounder concerning the role of elevated homocysteine plasma levels as a vascular risk factor. In two selected cohorts, we analysed MTHFR C677T;A1298C haplotypes and observed that likelihood of being a smoker was lowest in those subjects homozygous for the wild-type-alleles677CC and 1298AA, while presence of a mutant allele increased the likelihood of being a smoker. MTHFR is expressed in the brain and is involved in remethylation of homocysteine to methionine, which can be activated to S-adenosylmethionine functioning as methyl group donor, e.g. for the synthesis of neurotransmitters and dopamine methylation [9]. Whether an influence of MTHFR variants on such biochemical pathways may explain an association of these variants with smoking behaviour, remains speculative. Smoking is known to be associated with higher homocysteine plasma levels [5], [6]. Moreover, our data confirm that the T allele of MTHFR C677T and smoking interact in their association with homocysteine plasma levels, i.e. smoking homozygous T carriers had highest, non-smoking homozygous C carriers lowest homocysteine plasma levels [3].

Our study has several important limitations: Two heterogeneous study samples were retrospectively analysed. Both do not represent the normal population, but were recruited for studies on smoking cessation and atherosclerosis, respectively, which may have led to relevant selection bias as shown by the high rate of smokers. Controls did not completely match, thus, multivariate analyses were necessary. Further, such analysis of genotypes and smoking were initially not planned for sample 1, thus, information on smoking status was limited, i.e. “yes” versus “no” without clear definition. Further, we adjusted for age and sex, but other possible confounders like birth cohort effects and alcohol use were not regarded due to limited information. Results were only significant, if the two population samples were pooled, but only for trend or not in separate analyses, which would have been preferable due to the differences between those two samples. However, our data support to the theory that MTHFR C677T and smoking interact in generating elevated homocysteine plasma levels via unknown mechanisms. Such information may be relevant for the stratification of vascular risk factors. Moreover, our data suggest that MTHFR alleles with at least one of the mutant variants C677T and A1298C are associated with smoking. Published previous studies were inconclusive concerning such an association, which would be a relevant confounder concerning the association of MTHFR variants, hyperhomocysteinemia and vascular events. Speculatively, the difference between our findings – compared to prior negative results on associations between MTHFR genotype and smoking – may be due to analysing both variant regions C677T and A1298C simultaneously. Indeed, when C677T is analysed alone in our subjects, the difference between smokers and non-smokers was not significant. It may be justified that statistical strategies of future studies on the role of homocysteine metabolism in vascular disease regard such possible associations.
